# Primary HIV Drug Resistance among Recently Infected Cases of HIV in North-West India

**DOI:** 10.1155/2019/1525646

**Published:** 2019-02-27

**Authors:** C. K. Chauhan, P. V. M. Lakshmi, V. Sagar, A. Sharma, S. K. Arora, R. Kumar

**Affiliations:** ^1^Department of Community Medicine and School of Public Health, Post-Graduate Institute of Medical Education and Research, Chandigarh 160012, India; ^2^Department of Internal Medicine, Post-Graduate Institute of Medical Education and Research, Chandigarh 160012, India; ^3^Department of Immunopathology, Post-Graduate Institute of Medical Education and Research, Chandigarh 160012, India; ^4^Department of Epidemiology and Population Health, The London School of Hygiene and Tropical Medicine, London WC1E7HT, UK; ^5^School of Public Health and Community Medicine, University of New South Wales, Sydney 2033, Australia

## Abstract

**Background:**

Antiretroviral treatment may lead to the emergence of HIV drug resistance, which can be transmitted. HIV primary drug resistance (PDR) is of great public health concern because it has the potential to compromise the efficacy of antiretroviral therapy (ART) at the population level.

**Objective:**

To estimate the level of primary drug resistance among recently infected cases of HIV in 6 ART centres of North-Western India from September 2014 to June 2016.

**Methods:**

The level of primary drug resistance was studied among 37 recently infected HIV cases identified by Limiting antigen (Lag) avidity assay based on modified Recent Infection Testing Algorithm (RITA). The reverse transcriptase region of HIV-1* pol* gene (1-268 codons) was genotyped. The sequences were analyzed using the Calibrated Population Resistance (CPR) tool of Stanford University HIV drug resistance (DR) database to identify drug resistance.

**Results:**

Among 37 isolates studied, 6 (16.2%) samples showed primary drug resistance (PDR) against reverse transcriptase (RT) inhibitor. The proportion of primary drug resistance was 22.2% (2/9) among female sex workers, 14.3% (1/7) among men having sex with men, and 14.3% (3/21) among injecting drug users. Observed mutations were K219R, L74V, K219N, and Y181C. Injecting drug user (IDU) has showed resistance to either nucleoside/nucleotide reverse transcriptase inhibitors (NRTI) or nonnucleotide reverse transcriptase inhibitors (NNRTI).

**Conclusion:**

Resistance to either NRTI or NNRTI among the recently is a new challenge that needs to be addressed. The fact that both Y181C isolates are IDUs is important and represents 2/21 (~10%) NNRTI drug resistance. Surveillance for primary drug resistance (PDR) needs to be integrated into next generation of HIV surveillance as access to ART is increasing due to introduction of test and treat policy.

## 1. Introduction

ART is the main stay of treatment to delay the occurrence of AIDS. Nearly 9.7 million people were receiving antiretroviral therapy (ART) in low- and middle-income countries by the end of 2016 [1]. A mix of multiple antiretroviral drugs has been recommended to suppress HIV replication, thus preventing HIV linked mortality and morbidity apart from enhancing the quality of life of HIV/AIDS infected people. The introduction of zidovudine in 1987 started the era of ART but India started its antiretroviral therapy program for the treatment of HIV/AIDS in 2004; ARV drugs used in first line ART regimens for adults and adolescents in the national ART program in India are Zidovudine, Tenofovir, Abacavir, Lamivudine, Efavirenz, and Nevirapine [2]. The second-line ART regimens comprised of zidovudine (ZDV), lamivudine (3TC), tenofovir (TDF), and boosted lopinavir/ritonavir (LPV/r) have been introduced recently in a phase wise manner at various centres [3].

A major barrier to the long-term efficacy of ART is the emergence of drug resistant mutations in the polymerase gene of HIV-1, which reduces the susceptibility of the virus to antiretroviral (ARV) drugs. Factors that are associated with the development of drug resistance include use of monotherapy, inadequate suppression of viral replication, nonadherence to ART drugs, and initiation of therapy late in the course of HIV infection [4]. Because of fast rate of viral replication and lack of proof reading enzymes to correct errors occurring when virus converts its RNA into DNA via reverse transcription, HIV virus often makes mistakes in the copies, which may result in mutations and sometime mutations may lead to resistance against HIV drugs [5].

When resistant mutations emerge because of drug selective pressure in individuals receiving antiretroviral therapy it is known as acquired resistance. On the other hand primary drug resistance occurs when previously uninfected individuals are infected with a drug resistant virus; transmitted drug resistance can also occur from people with transmitted drug resistance, i.e., secondary transmission [5]. There is an increasing trend in the prevalence of primary drug resistance from 1.1% to 21% in the United States, Africa, and Europe [6–12].

Currently, HIV drug resistance testing prior to ART initiation is not being routinely performed in resourced-limited countries like India, due to lack of infrastructure. Earlier studies have reported low prevalence of HIV drug resistance in India and these studies are now 5-10 years old [13–17]. Earlier studies on drug resistance are useful in providing knowledge about the pattern and trend of drug resistance. Thus objective of the present study was to estimate the level of primary drug resistance by genotyping method among recent cases of HIV-1 infected individuals among HRGs from North-West India.

## 2. Material and Methods

### 2.1. Patient Selection

Forty newly diagnosed HIV positive patients identified from 64, consecutively recruited individuals, who visited ART centres for evaluation of their CD4 count, were recruited in this study, from September 2014 to June 2016. Selection criteria were to select HIV positive individuals among HRGs registered for pre-ART in last one year at ART centres of North-West India, who have not received any kind of antiretroviral therapy prior to enrollment in the study, who were ≥ 18 years old, and whose CD4+ count was ≥200 cells/*μ*l. Such patients were included for identifying incident cases of HIV-1 using modified RITA based on Limiting antigen (Lag) avidity assay [18, 19]. Patients on Directly Observed Treatment, Short Course (DOTS) for Tuberculosis were excluded from study. Prior permission was obtained from National AIDS Control Organization (NACO) and respective State AIDS Control Society (SACS) before starting the study. ART centre in-charge, counsellor, and laboratory technician were informed regarding the study by the State AIDS Control Society (SACS). Written informed consent was obtained from all the participants before enrollment. Study participants were interviewed using semistructured questionnaire and information about sociodemographic characteristics, history of sexual, and other behavioural risk factors and treatment history especially intake of antiretroviral drugs and CD4 cell counts was collected.

### 2.2. Specimens Collection

From each selected individual, 5 ml of venous blood was collected in a K_3_EDTA vacutainer tube (Becton Dickinson, USA) by the Lab. Technician. Out of the 5ml, 2ml blood was used for CD4 count estimation at respective ART centres. The remaining 3ml blood was centrifuged in a bench top centrifuge within the vacutainer vial, at 2500 rcf for 10 minutes at room temperature for obtaining the plasma. The plasma was aspirated using a Pasteur pipette and stored in separate vials at -80°C for determination of recent infection. The recent cases identified using RITA were genotyped for studying the primary drug resistance.

### 2.3. Drug Resistance Genotyping

For drug resistance genotyping, viral RNA was extracted from 560 *μ*l of plasma using the QIAamp Viral RNA Mini kit (Qiagen, Valencia, CA, United States) according to the manufacturer's instructions. RNA was recovered from the spin columns in a final elution volume of approximately 60 *μ*l. The HIV-1* pol *gene (reverse transcriptase 1–268 codon) was amplified using nested reverse transcription polymerase chain reaction (RT-PCR) method. The target sequence was amplified with using One-Step Invitrogen Super Script III RT-PCR System with Platinum Taq DNA Polymerase (Invitrogen Life Technologies, Carlsbad, CA) using primers* pol* RT F 5'-TTC CCA TTA GTC CTA TTG AAA CTG T-3' and* pol *RT R 5'-TCA TTG ACA GTC CAG CTA TCC TTT T-3' in a 25 *μ*l reaction. Cycling conditions were 45°C for 40 min and 94°C for 1 min in first-round RT-PCR, followed by 30 cycles at 94°C for 1 min, 58°C for 1 s,72°C for 1min, and an extension at 72°C for 10 min. The nested PCR was performed using Super Script III Taq Platinum PCR kit using primers* pol *RT R 5′-CAG AGC CAA CAG CCC CAC CA-3′ and* pol *RT F 5'- GCC TGA AAA TCC ATA TAA CAC TCC -3′ in a 25 *μ*l reaction and the cycling conditions were 95°C for 5 min in first-round RT-PCR, followed by 35 cycles at 94°C for 30s,58°C for 30 s,72°C 2.5 min, and an extension at 72°C for 10 min. PCR purification was done using Sure Extract Spin PCR Clean-up/Gel Extraction kit (Genex Biotech, New Delhi, India). The PCR purified product was detected and analyzed on 1% agarose gel. PCR products corresponding to the RT coding regions were sequenced.

Purified product (15-45ng) was used for sequencing, using forward primer and big dye terminator ready reaction mix (ABI Big Dye Terminator version 3.0 Ready Reaction Cycle Sequencing Kit, Applied Biosystems, USA) according to the manufacturer's instructions. For sequencing reaction temple (15-45ng), forward primer 5'- GCC TGA AAA TCC ATA TAA CAC TCC-3' reverse primer 5'- CCA TCC AAA GAA ATG GAG GTT C-3' (1.6*μ*l of 10pmol concentration) of the* pol* gene, PCR buffer (1.5*μ*l), and water (variable) were added to 2-4*μ*l ABI Prism Big Dye (which contains DNA polymerases, dNTPs, and labeled ddNTPs). 10*μ*l of the reaction was set up for sequencing and the samples were subjected to thermocycler GeneAmp® PCR system 9700 (Applied Biosciences, USA). The reaction mixture was brought at 4°C and then at -20°C till further use. Purification of the sequencing reaction mix, i.e., removal of the unincorporated dye, unused primer, and unused dNTPs, was carried out according to version used. The samples were then loaded on to the sequencing machine, 3130xl genetic analyzer (Applied Biosystems, USA). Sequencing was done according to manufacturer's instructions.

In present study RT region alone was genotyped. Due to fund constraints we could focus only on RT region. The* pol *(RT region) gene sequences were submitted to the Stanford University HIV Drug Resistance Database (hivdb.stanford.edu/) version 6.0.11. Primary drug resistance and HIV subtype were determined according to the Stanford HIV database [20].

Quality Control Procedure. For quality control of HIV-1 sequencing, low-positive and high-positive control samples were run with every batch. The positive controls ensured the RT-PCR and sequencing success. To ensure good sequence quality, the high-positive control was sequenced precluding editing mistakes. Phylogenetic analysis was performed to check for contamination as per procedures described in the Los Alamos website (http://www.hiv.lanl.gov/).

### 2.4. Clade Typing and Phylogenetic Tree

HIV subtype was determined according to the Stanford HIV database [20]. Phylogenetic tree was constructed with the neighbor-joining method within the Molecular Evolutionary Genetics Analysis program (MEGA) version 6 after multiple alignments of data by CLUSTAL X. Pairwise evolutionary distances were estimated using Kimura's two parameter method and reliability of the topologies was estimated by performing bootstrap analysis (1000 replicates). Thirty-three known reference sequences were included from India, Nepal, China, Myanmar, South Africa, Norway, Ethiopia, Swaziland, Zimbabwe, Botswana, and United Kingdom which were obtained from Los Alamos National Laboratory HIV sequence database of common HIV-1 subtype C; HIV-1 subtype K from United State of America was used as an outgroup species.

## 3. Results

**Figure 1:**
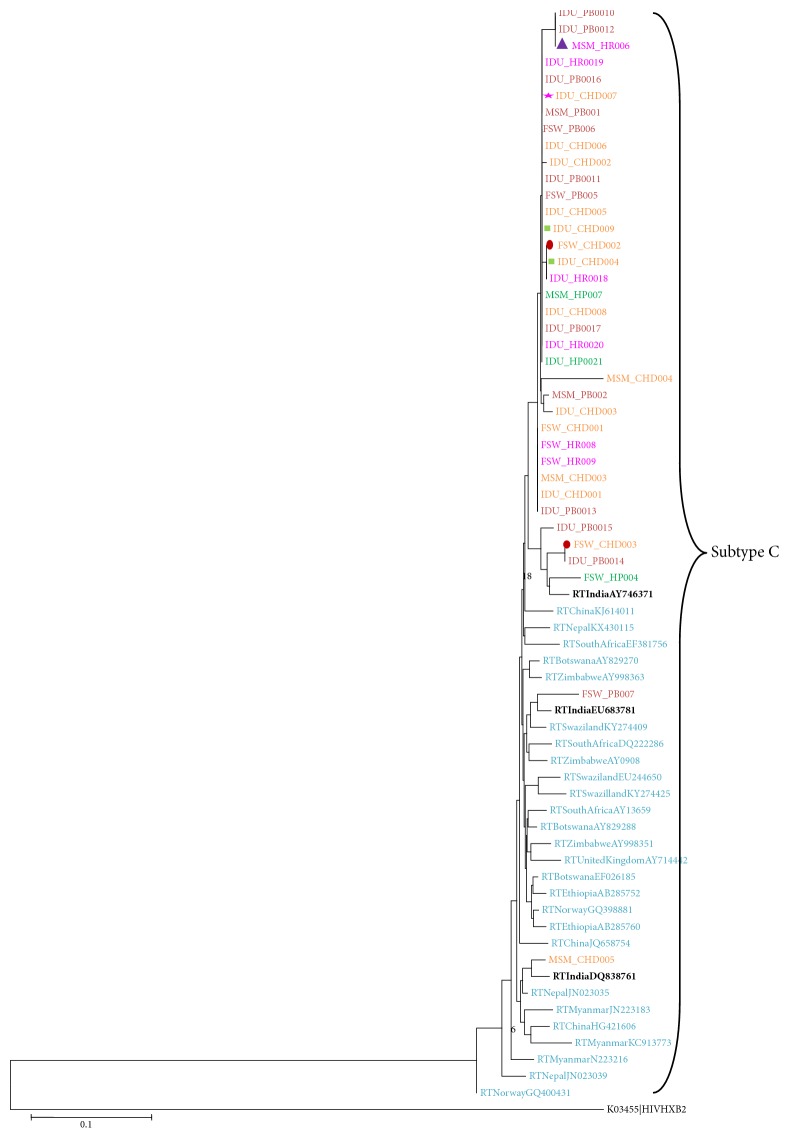
Phylogenetic relationship based on* pol *gene (reverse transcriptase 1–268 codon) sequences. Sequences are from Punjab shown in (dark red color), Haryana (pink color), Chandigarh (Brick red color), and Himachal Pradesh (Green color). Drug resistance isolates highlighted in different symbols, i.e., ∆: L74V, ★: K219N, □: Y181C, *⬯*: K219R. Global HIV-1 subtype C reference sequences (shown in blue & black color) and K obtained from Los Alamos HIV Sequence Database. Both Y181C isolates (IDU_CHD004 and IDU_CHD009) are IDUs; they are unique and not showing any clustering or interlinking.

Out of 64 recently infected individuals, only 37 samples (who had CD4 count between 200 and 500 cells/*μ*l) could be genotyped. Of the total 37 samples, 9 were female sex worker (FSW), 7 were men who have sex men (MSM), and 21 were injecting drug user (IDU) samples. The mean age of the respondents was 34.8 (SD=10) years. The median CD4 count was 332.5 cells/*μ*l (IQ range 257-491). All 37 samples belonged to subtype C (Figure 1)

Out of 37 samples sequenced for HIV-1* pol* gene (RT region) 6 isolates (FSW: 2, MSM: 1, and IDU: 3) showed primary drug resistance against RT inhibitors (Stanford HIV RT sequence database), thus prevalence of primary drug resistance was 16.2% (95% CI: 6.8-30.7). The proportion of primary drug resistance was 22.2% (95% CI: 3.9-56) among FSWs, 14.3% (95% CI: 0.7-53.2) among MSMs, and 14.3% (95% CI: 3.7-34.1) among IDUs. Confidence interval of all 3 risk groups was overlaps. This signifies that there was no significant difference across the groups. Out of 6 resistant isolates, 3 had a NRTI resistance mutation (K219R:2; K219N:1) that is known to cause potential low level resistance to Zidovudine (AZT). Out of these 3 isolates, 2 belonged to FSW; 1 belonged to IDU. One isolate, belonging to MSM, had L74V, a NRTI resistance mutation that is known to cause Intermediate Resistance to Abacavir (ABC). Two isolates had Y181C, a major NNRTI resistance mutation accountable for high level resistance to Nevirapine (NVP), intermediate resistance to efavirenz (EFV), etravirine (ETR), and rilpivirine (RPV). Both of these isolates belonged to IDU (Table 1).

## 4. Discussion

Drug resistance is a major challenge for achieving viral suppression. In this study, we have sequenced the amplified RT region of* pol* gene of HIV isolates from recently infected and treatment naïve high risk individuals in north-west India. Primary drug resistance mutations were identified as per Stanford DR database. In India first line of ART comprised of RTI drugs and a higher rate of DR mutations have been reported in the RT region in therapy naïve individuals in India [21]. The most common subtype of HIV-1 in India is subtype C [17, 22]. In the present study primary drug resistance was observed 16.2% (95% CI: 6.8-30.7) recently infected individuals. According to WHO, the drug resistance prevalence in a geographical area can be categorized as <5%, 5–15% and >15% [23]. Thus primary drug resistance in our study can be categorized as moderate to high prevalence according to World Health Organization (WHO) criteria.

Earlier report of primary drug resistance (PDR) varied between 0% and 13.8% in Asian countries. Primary drug resistance in individuals who have not started receiving treatment but are infected with HIV-1 subtype B or its recombinants reported from USA and the Western Europe ranges from 10% to 17% [24]. Studies on drug resistance from Central America and Mexico found that the PDR rates among adults were variable. It ranges from as low as 0% in Panama to as high as 12.5% in certain areas of Mexico [25, 26]. The El Salvador reported a prevalence of 5.7% in the general HIV-infected population which is lower in comparison to the neighboring nations like Honduras and Guatemala [27]. PDR rate was high (12%) among studies done on the paediatric HIV-infected population stressing on urgent requirement of more such studies assessing PDR in these population [28, 29].

Among different countries, the prevalence of primary drug resistance in its decreasing order is Australia (17.5%), followed by France (16.7%), whereas USA (12.6%) stands equal to Spain (12.6%) [30]. Earlier studies showed the global PDR prevalence among different continents in the order of Africa (4.7%), Latin America (6.3%), Europe (10.9%), and North America (12.9%). The prevalence of PDR has significantly increased in past few years due to ART becoming more available in these continents [31, 32]. In these settings, the use of mono and dual therapies in the highly active antiretroviral therapy (HAART) era, sequential functional monotherapy, and the use of suboptimal regimens in the early HAART era and ongoing difficulties with adherence and tolerability have led to the accumulation of drug resistance in treatment-experienced patients and the subsequent spread of PDR [33].

In recent studies in India, the prevalence of primary drug resistance has ranged between 0% and 6.7% [14–18]. All previous studies were at least 4-5 years old. During this period ART use has increased from 36% to 50% and this increase could explain a higher level of primary drug resistance observed by present study.

Comparatively higher prevalence of primary drug resistance (PDR) was found in key population who are recently infected than those found in earlier surveys of primary drug resistance (PDR) in Asia [2]. A prevalence of 7% for primary resistance was reported in Thailand for men who have sex with men [34]. Study conducted by Murillo* et al.* (2012) in Central America concluded that the prevalence of PDR among female sex workers and men who have sex with men was 10.3% and 9%, respectively [35]. Higher prevalence found in the present study could be because present study was conducted among high risk groups who already have higher HIV prevalence than general adult populations. India has a concentrated epidemic among FSW (1.56%, 95% CI: 1.46-1.66), MSM (2.69%, 95% CI: 2.47-2.91), and IDU (6.26%, 95% CI: 5.92-6.59) [36]. While HIV prevalence among the general population is 0.3%. So far no study has been conducted in North-West India which could measure the PDR among HRGs. Study conducted by Iqbal* et al. *(2009) in South India on Injecting Drug Users (IDUs) showed that the PDR was 2.9% [37].

In the present study the prevalence of primary drug resistance against antiretroviral drug was found to be moderate whereas earlier studies from India on primary drug resistance have shown lower level of prevalence [13–17]. This may be due to difference in various factors, namely, study methodologies, route of HIV-1 transmission, period of infection, HIV-1 subtype, ART regimen use, and the reference list of resistance analogue Mutations (RAMs) used to evaluate the presence of relevant mutations. Limitation of study is the small sample size and only RT region was sequenced, as HIV genotyping is costly, and limited funding was available for the study. Despite these limitations the present study does reflect the rise in drug resistance in North-West India, which deserves attention.

## 5. Conclusion

In conclusion, our study indicates that a moderate to high level of primary drug resistance was focused after the rapid expansion of ART program in India. Primary drug resistance was detected among 16% treatment naïve HRGs in north-west India by genotyping assay. The treatment of HIV has become increasingly complex with the introduction of new ARVs drugs and need of the hour is to understand ARV drug resistance development in order to effectively combat HIV infections. Resistance to NNRTI among IDUs is a new challenge that needs to be addressed. National programs to monitor HIV drug resistance among HIV-infected population should be done through HIV genotyping in sentinel populations.

## Figures and Tables

**Table 1:** Drugs affected by mutations detected in six of the thirty-seven recently HIV positive individuals in RT-region of *pol* gene.

High Risk group/State	AGE& gender	NRTI	NNRTI	Drugs Affected
MSM Haryana	26/Male	L74V		Intermediate Resistance: Abacavir ( ABC)

IDU Chandigarh	44/Male	K219N		Potential Low Level Resistance: Zidovudine (AZT)

FSW Chandigarh	25/Female	K219R		Potential Low Level Resistance: Zidovudine (AZT)

FSW Chandiagrh	38/Female	K219R		Potential Low Level Resistance: Zidovudine (AZT)

IDU Chandigarh	21/Male		Y181C	High-Level resistance: Nevirapine (NVP)
				Intermediate Resistance: efavirenz (EFV), etravirine (ETR), rilpivirine (RPV)

IDU Chandigarh	32/Male		Y181C	High-Level resistance: Nevirapine (NVP)
				Intermediate Resistance: efavirenz (EFV), etravirine (ETR), rilpivirine (RPV)

## Data Availability

The primers used in the present are included within the article.

## Additional Points

*Sequence Data. *RT nucleotide sequences of study isolates are submitted to gene bank under the submission numbers BankIt2182117 (5), BankIt2182140 (5), BankIt2182149 (5), BankIt2182158 (5), BankIt2182183 (5), BankIt2182193 (5), BankIt2182199 (5), and BankIt2182205 (2).
